# Genetic Characterization and Zoonotic Potential of *Cryptosporidium* spp. and *Giardia duodenalis* in Cattle From Northeast China

**DOI:** 10.1155/tbed/6148130

**Published:** 2025-07-24

**Authors:** Qile Yu, Sining Chen, Xichen Zhang, Qi Zhao, Mengfei Xu, Jianhua Li, Pengtao Gong, Xiaocen Wang, Xin Li, Xu Zhang, Nan Zhang

**Affiliations:** ^1^ State Key Laboratory for Diagnosis and Treatment of Severe Zoonotic Infectious Diseases, Key Laboratory for Zoonosis Research of the Ministry of Education, Institute of Zoonosis, and College of Veterinary Medicine, Jilin University, Changchun, 130062, China, jlu.edu.cn

**Keywords:** *cryptosporidium* spp., genotypes, *giardia duodenalis*, prevalence, subtypes

## Abstract

*Cryptosporidium* spp. and *Giardia duodenalis* (*G. duodenalis*) are zoonotic and gastrointestinal parasites infecting humans and animals. This study investigated the prevalence and subtypes of *Cryptosporidium* and *G. duodenalis* in cattle from six farms across three provinces and one autonomous region in northeast China. Fecal samples (*n* = 495) were detected using PCR‐based methods: *Cryptosporidium* was genotyped via PCR‐restriction fragment length polymorphism (RFLP) targeting the small subunit ribosomal RNA (*SSU* rRNA) gene, and *G. duodenalis* assemblages were identified through multilocus sequence typing of the *β*‐giardin (*bg*), glutamate dehydrogenase (*gdh*), and triose phosphate isomerase (*tpi*) genes. Overall infection rates were 44.44% (220/495) for *Cryptosporidium* and 39.39% (195/495) for *G. duodenalis*. Age‐related trends showed *Cryptosporidium* prevalence peaked in preweaned calves (63.29%, 50/79), whereas the highest prevalence rate of *G. duodenalis* infections (53.66%, 66/123) was observed in calves aged 3–11 months. Four *Cryptosporidium* species (*C. parvum*, *C. bovis*, *C. ryanae*, and *C. andersoni*) and coinfections involving two or three *Cryptosporidium* species were identified. Based on 60‐kilodalton glycoprotein (*gp60*) gene of *C. parvum*, three subtypes (IIdA19G1, IIdA24G2, and IIdA21G1) were identified. Subtype IIdA21G1 was reported for the first time in cattle, with its initial detection occurring in China. For *G. duodenalis*, assemblages A and E were identified in all four areas, with 13 assemblage E multilocus genotypes (MLGs), one assemblage A MLG, and eight mixed (A + E) MLGs detected. Our findings revealed a novel genetic subtype of *C. parvum* in China, and the high prevalence of both *Cryptosporidium* and *G. duodenalis* suggested an increased zoonotic risk that deserved more attention.

## 1. Introduction

*Cryptosporidium* spp. and *Giardia duodenalis* (*G. duodenalis*) are significant protozoan parasites capable of infecting a wide range of vertebrate hosts [1–3]. Both parasites cause gastrointestinal diseases characterized by diarrhea, malnutrition, and immunosuppression in humans and animals, posing substantial public health risks [4, 5]. Cryptosporidiosis and giardiasis exhibit a global distribution, with a higher prevalence in developing and resource‐limited regions [6]. Infections in immunocompromised populations, including children, the elderly, and individuals with immunodeficiency, can lead to severe health consequences [7, 8].

Cryptosporidiosis and giardiasis can cause substantial economic losses to the livestock industry, particularly in cattle farming [9]. Cases of bovine cryptosporidiosis and giardiasis have been reported worldwide [10, 11]. While both parasites are generally nonfatal to adult cattle, they pose a serious threat to calves, especially those are preweaned [12, 13]. Infection in preweaned calves can lead to symptoms such as diarrhea, loss of appetite, lethargy, fever, and severe dehydration, and in severe cases, even death [14, 15]. Both *Cryptosporidium* and *G. duodenalis* are transmitted via the fecal–oral route [16]. Once oo/cysts are released into the environment, they could cause widespread infection [17, 18].

The identification of species, genotypes, and subtypes of *Cryptosporidium* and *G. duodenalis* is critically important for elucidating the pathogens and their transmission sources. To date, at least 44 valid *Cryptosporidium* species and more than 120 genotypes have been characterized [3]. Studies have shown that the most prevalent *Cryptosporidium* species in cattle include *C. bovis*, *C. parvum*, *C. andersoni*, and *C. ryanae* [19–21]. Among these, *C. parvum* is primarily associated with infections in preweaned calves, whereas the other three species are more frequently detected in adult cattle [22, 23]. Currently, conventional PCR [24, 25], nested PCR [26, 27], and quantitative PCR [28, 29] are routinely employed for the molecular identification and characterization of *Cryptosporidium*. By targeting variable regions and sequence polymorphisms in key genetic markers, such as the small subunit ribosomal RNA (*SSU* rRNA), acetyl‐CoA synthetase (ACS), heat shock protein 70 (HSP70), 60‐kilodalton glycoprotein (*gp60*), and *Cryptosporidium* oocyst wall protein (COWP) genes [30–32], these molecular approaches enable accurate differentiation among various species and subspecies. For *Giardia*, genotyping is primarily based on sequence analysis of key genetic loci, including the small subunit ribosomal RNA (*SSU* rRNA), *β*‐giardin (*bg*), glutamate dehydrogenase (*gdh*), and triose phosphate isomerase (*tpi*) genes [33–36]. Using multiple genetic markers significantly improves the reliability of genotyping results and facilitates the identification of mixed infections with diverse genetic combinations. *G. duodenalis* is classified into eight distinct assemblages (A‐H) [37, 38], which exhibit minimal morphological differences but demonstrate significant host specificity. Assemblages A and B of are known to infect humans and a wide range of mammalian species [39], highlighting their substantial zoonotic potential. Assemblages C and D primarily infect canids, while assemblage E is predominantly associated with infections in pigs and ruminants [6]. Assemblage F is commonly found in felids, assemblage G is restricted to rodents [40], and assemblage H exclusively infects marine mammals [41].

The scale of animal husbandry and farming models is predominantly industrialized in northeast China. In 2023, there were ~440,000 animal husbandry enterprises, accounting for 16.13% of the national total [42]. Concurrently, the potential risks for outbreaks of cryptosporidiosis and giardiasis in cattle enterprises rise with the expansion of the animal husbandry sector in northeast China. Furthermore, the unique climatic conditions of northeast China, characterized by low temperatures for significant portions of the year, are particularly relevant to the environmental transmission of protozoan parasites. Critically, low‐temperature environments have been shown to significantly prolong the environmental persistence of oo/cysts [6, 43]. Epidemiological studies on *Cryptosporidium* and *Giardia* have been extensively conducted in numerous farming regions across China [44]. However, comprehensive data from northeastern China remain scarce. Therefore, this study aims to investigate the prevalence, species distribution, genotypes, and subtypes of *Cryptosporidium* and *Giardia* in cattle populations in northeastern China. The results will provide a scientific basis for formulating targeted prevention and control measures. Additionally, by assessing potential genetic variations in both pathogens, this study will offer a comprehensive evaluation of their interspecies and cross‐species transmission potential, particularly in the context of zoonotic risks.

## 2. Materials and Methods

### 2.1. Fecal Sample Collection

**Figure 1:**
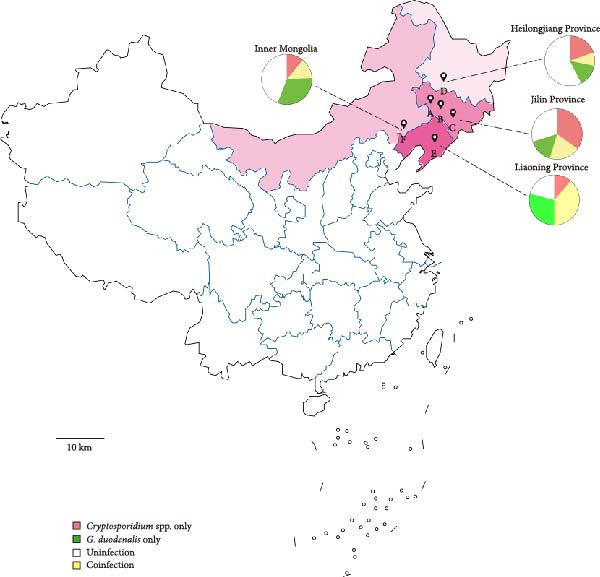
Geographical locations of sample collection sites. The “
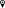
” marker represents the geographical sampling locations of this study. The pie chart illustrates the infection proportions of *Cryptosporidium* spp. and *G. duodenalis* across different regions.

From May to June 2024, a total of 495 fecal samples were randomly collected from six cattle farms situated across three provinces and one autonomous region in northeast China (Figure 1), with the following distribution: 86 samples from Farm A, 93 samples from Farm B, 79 samples from Farm C, 92 samples from Farm D, 62 samples from Farm E, and 83 samples from Farm F. Each sample was collected from the rectum of each cattle with new disposable gloves, which was dispensed in a new 50 mL centrifuge tube with a recorded number. Immediately, these 50 mL centrifuge tubes with fresh fecal samples were transported into the ice box. Fecal samples processing, including genomic DNA extraction and molecular identification, were completed within 24 h.

### 2.2. Genomic DNA Extraction

Genomic DNA was extracted from ~200 mg of each solid fecal sample or 200 μL of liquid fecal sample using the TIANamp Stool DNA kit (TIANGEN, Beijing, China) with additional procedures. Specifically, fecal samples were first resuspended in lysis buffer (buffer SA and SC from the kit) containing proteinase K. This was followed by five cycles of freezing in liquid nitrogen for 1 min and thawing at 37°C for 4 min. The subsequent steps were performed according to the manufacturer’s instructions. The extracted genomic DNA was stored at −20°C until further use.

### 2.3. PCR Amplification

*Cryptosporidium* spp. were identified via nested PCR amplification of *SSU* rRNA gene [45]. Subtyping of *C. parvum* was performed by nested PCR amplification targeting the *gp60* gene [45]. The *bg*, *gdh*, and *tpi* genes of *G. duodenalis* were amplified for molecular identification and multilocus genotyping [33, 46, 47]. Primers, conditions, and product sizes of all PCRs were listed in Table S1. The amplification reaction mixture of first PCR consisted of 25 μL of 2 × Premix Taq DNA Polymerase (Takara Bio Inc., Dalian, China), 1 μL of each primer (10 μM), and 100 ng genomic DNA. In the secondary PCR step, 1 µL of the primary PCR product was used as the template. The concentrations of the other components were consistent with those used in the first PCR. Each reaction included three controls: a positive control (DNA extracted from the positive fecal sample), a negative control (DNA extracted from the negative fecal sample), and a blank control (deionized water). Amplification products were visualized under UV light after electrophoresis in a 2% agarose gel containing nucleic acid gel stain (Yeasen Biotech, Shanghai, China).

### 2.4. RFLP Analysis

For the analysis of restriction fragments, a 20 μL reaction mixture containing 16 μL of the secondary PCR products of the *SSU* rRNA gene, 10 U of SspI‐HF (New England BioLabs, Beverly, Mass.) or 10 U of MboII (New England BioLabs, Beverly, Mass.), and 2 μL of rCutSmart Buffer was incubated at 37°C for 1 h. The restriction digestion products were separated by electrophoresis on 2% agarose gels. The results provided robust support for the identification of *Cryptosporidium* species and concurrent infections [45]. For coinfection samples, PCR and restriction fragment length polymorphism (RFLP) verification would be performed three times to avoid false positives, followed by sequencing analysis.

### 2.5. Sequence and Phylogenetic Analysis

All positive products were sequenced bidirectionally on an ABI 3730xL Genetic Analyzer (Applied Biosystems, CA, USA). The sequences we obtained were identified by aligning the sequences with known reference sequences from GenBank using the BLAST tool (http://www.ncbi.nlm.nih.gov/BLAST/), and phylogenetic analysis was constructed in MEGA 11 (http://www.megasoftware.net/) using the neighbor‐joining (NJ) method implemented with evolutionary distances calculated by the best‐fitting model to describe a robust estimate of the evolutionary distances.

### 2.6. Statistical Analysis

Statistical analyses were conducted using SPSS 19.0 (IBM Corp., Armonk, NY), with Chi‐square (*χ*2) analysis and 95% confidence intervals (CIs) applied to evaluate geographic and age‐stratified differences, adopting a significance threshold of *p* = 0.05. For age‐stratified analysis, Fisher’s exact test was used instead of the *χ* ^2^ test for the >2‐year‐old group (*n* = 41, with 4 positives) because expected frequencies were <5.

## 3. Results

### 3.1. Prevalence of Cryptosporidium spp. and *G. duodenalis*

A total of 495 fecal samples were collected from six cattle farms across three provinces and one autonomous region in northeast China. Specimens were distributed across developmental stages as follows: 207 from preweaned calves (<3 months), 205 from postweaning calves (3–11 months), 42 from juvenile cattle (1–2 years), and 41 from mature cattle (>2 years). PCR analysis revealed a high prevalence of both parasites, with *Cryptosporidium* detected in 44.44% (220/495, 95% CI 40.1–48.8) and *G. duodenalis* in 39.39% (195/495, 95% CI 35.1–43.7) (Tables 1 and 2). Notably, all six farms tested positive for both parasites.

**Table 1:** Occurrence and genotype of *Cryptosporidium* spp. in cattle in northeast China.

Factor	Farm ID	Sample size	No. positive	Prevalence (%) (95% CI)	*p* Value	*Cryptosporidium* species (No.)
Location						
Jilin	Farm A	86	28	32.56 (22.5–42.7)	—	*C. parvum* (20), *C. bovis* (5), *C. andersoni* (1), *C. ryanae* (2)
	Farm B	93	62	66.67 (56.9–76.4)	0.30	*C. parvum* (37), *C. bovis* (15), *C. andersoni* (5), *C. ryanae* (4), *C.bovis* + *C. ryanae* (1)
	Farm C	79	51	63.29 (53.8–75.3)	0.02	*C. parvum* (40), *C. bovis* (3), *C. andersoni* (2), *C. ryanae* (2), *C.bovis* + *C. ryanae* (2), *C. parvum* + *C. ryanae* (1), *C.bovis* + *C. parvum* (1)
Sub‐total	—	258	141	54.65 (48.5–60.8)	<0.01	*C. parvum* (97), *C. bovis* (23), *C. andersoni* (8), *C. ryanae* (8), *C.bovis* + *C. ryanae* (3), *C. parvum* + *C. ryanae* (1), *C.bovis* + *C. parvum* (1)
Heilongjiang	Farm D	92	26	28.26 (18.9–37.6)	0.58	*C. parvum* (16), *C. ryanae* (1), *C. parvum* + *C. ryanae* (1), *C.bovis* + *C. ryanae* (3), *C. parvum* + *C.bovis* + *C. ryanae* (5)
Liaoning	Farm E	62	31	50.00 (37.2–62.8)	<0.01	*C. parvum* (1), *C. bovis* (10), *C. ryanae* (18), *C.bovis* + *C. ryanae* (2)
Inner Mongolia	Farm F	83	22	26.51 (16.8–36.2)	—	*C. parvum* (10), *C. bovis* (10), *C. andersoni* (1), *C. parvum + C. bovis* (1)
Age						
Preweaned		207	131	63.29 (56.7–69.9)	<0.01	*C. parvum* (121), *C. ryanae* (9), *C. parvum* + *C. ryanae* (1)
3–11 months		205	79	38.54 (31.8–45.3)	<0.01	*C. parvum* (3), *C. ryanae* (18), *C. bovis* (41), *C. andersoni* (1), *C. parvum* + *C. ryanae* (1), *C.bovis* + *C. andersoni* (1), *C.bovis* + *C. ryanae* (7), *C. parvum+ C. bovis* (2), *C. parvum* + *C.bovis* + *C. ryanae* (5)
1–2 years		42	6	14.29 (3.2–25.3)	0.93	*C. bovis* (2), *C. andersoni* (4)
> 2 years		41	4	9.76 (0.3–19.2)	—	*C. andersoni* (4)
Total		495	220	44.44 (40.1–48.8)	—	—

**Table 2:** Occurrence and assemblages of *G. duodenalis* in cattle in northeast China.

Factor	Farm ID	Sample size	No. positive	Prevalence (%) (95% CI)	*p* Value	No. positive (%)			Assemblage (No.)		
						bg	gdh	tpi	bg	gdh	tpi
Location											
Jilin	Farm A	86	40	46.51 (35.8–57.3)	0.05	32 (37.21)	18 (20.93)	12 (13.95)	E (27), A (5)	E (17), A (1)	E (7), A (5)
	Farm B	93	29	31.18 (21.6–40.8)	<0.01	15 (16.13)	20 (21.51)	11 (11.83)	E (11), A (4)	E (16), A (5)	E (9), A (2)
	Farm C	79	22	27.85 (17.7–38.0)	—	13 (16.46)	11 (13.92)	15 (18.99)	E (12), A (1)	E (11)	E (8), A (7)
Sub‐total	—	258	91	35.27 (29.4–41.1)	<0.01	60 (23.26)	49 (18.99)	38 (14.73)	E (50), A (10)	E (43), A (6)	E (24), A (14)
Heilongjiang	Farm D	92	21	22.83 (14.1–31.6)	—	12 (13.04)	11 (11.96)	13 (14.13)	E (2), A (10)	E (2), A (9)	E (13)
Liaoning	Farm E	62	42	67.74 (55.8–79.7)	<0.01	31 (50.00)	30 (48.39)	23 (37.10)	E (30), A (1)	E (30)	E (14), A (9)
Inner Mongolia	Farm F	83	41	49.40 (38.4–60.4)	<0.01	33 (39.76)	32 (38.55)	10 (12.05)	E (28), A (5)	E (27), A (5)	E (9), A (1)
Age											
Preweaned	—	207	76	36.71 (30.1–43.3)	<0.01	50 (24.15)	40 (19.32)	40 (19.32)	E (35), A (15)	E (32), A (8)	E (27), A (13)
3–11 months	—	205	110	53.66 (46.8–60.5)	<0.01	83 (40.49)	77 (37.56)	40 (19.51)	E (72), A (11)	E (66), A (11)	E (30), A (10)
1–2 years	—	42	7	16.67 (4.9–28.4)	0.09	1 (2.38)	4 (9.52)	3(7.14)	E (1)	E (3), A (1)	E (2), A (1)
> 2 years	—	41	2	4.88 (0–11.8)	—	2 (4.88)	1 (2.44)	1(4.88)	E (2)	E (1)	E (1)
Total	—	495	195	39.39 (35.1–43.7)	—	136 (27.47)	122 (24.65)	84 (17.00)	E (110), A (26)	E (102), A (20)	E (60), A (24)

*Cryptosporidium* prevalence exhibited significant geographical variation across the surveyed farms (*χ* ^2^ = 32.236, *df* = 3, *p*  < 0.01), with farms from Jilin Province showing the highest prevalence (54.65%, 95% CI 48.5–60.8), followed by Liaoning (50.00%, 95% CI 37.2–62.8), Heilongjiang (28.26%, 95% CI 18.9–37.6), and Inner Mongolia (26.51%, 95% CI 16.8–36.2). In Jilin, a total of 258 fecal samples were collected from three cattle farms (A, B, and C). Farm‐level analysis revealed striking disparities in infection rates (*χ* ^2^ = 25.483, *df* = 2, *p* < 0.01): Farm B demonstrated the highest *Cryptosporidium* prevalence (66.67%, 95% CI 56.9–76.4), followed by Farm C (63.29%, 95% CI 53.8–75.3) and Farm A (32.56%, 95% CI 22.5–42.7). Furthermore, infection risk displayed a strong age‐dependent pattern. The highest prevalence was observed in preweaned calves (63.29%, 95% CI 56.7–69.9), progressively decreasing with advancing age: 3–11 months (38.54%, 95% CI 31.8–45.3), 1–2 years (14.29%, 95% CI 3.2–25.3), and >2 years (9.76%, 95% CI 0.3–19.2) (*p* < 0.01).

*G. duodenalis* infection displayed a distinct geographic gradient across the study regions, highlighting province‐level differences in exposure risks (*χ* ^2^ = 36.761, *df* = 3, *p* < 0.05). The Farm E from Liaoning Province demonstrated the highest prevalence (67.74%, 95% CI 55.8–79.7), significantly exceeding rates in Inner Mongolia (49.40%, 95% CI 38.4–60.4), Jilin (35.27%, 95% CI 29.4–41.1), and Heilongjiang (22.83%, 95% CI 14.1–31.6). Within Jilin Province, substantial farm‐level variation was observed (*χ* ^2^ = 7.347, *df* = 2, *p* < 0.05). Farm A recorded the highest positivity rate (46.51%, 95% CI 35.8–57.3), followed by Farm B (31.18%, 95% CI 21.6–40.8) and Farm C (27.85%, 95% CI 17.7–38.0). Age stratification revealed a distinct epidemiological pattern (*p* < 0.01): prevalence peaked in calves aged 3–11months (53.66%, 95% CI 46.8–60.5) and preweaned calves (36.71%, 95% CI 30.1–43.3), followed by a progressive decline in cattle aged 1–2 years (16.67%, 95% CI 4.9–28.4) and those >2 years (4.88%, 95% CI 0–11.8).

### 3.2. *Cryptosporidium* Genotypes

**Figure 2:**
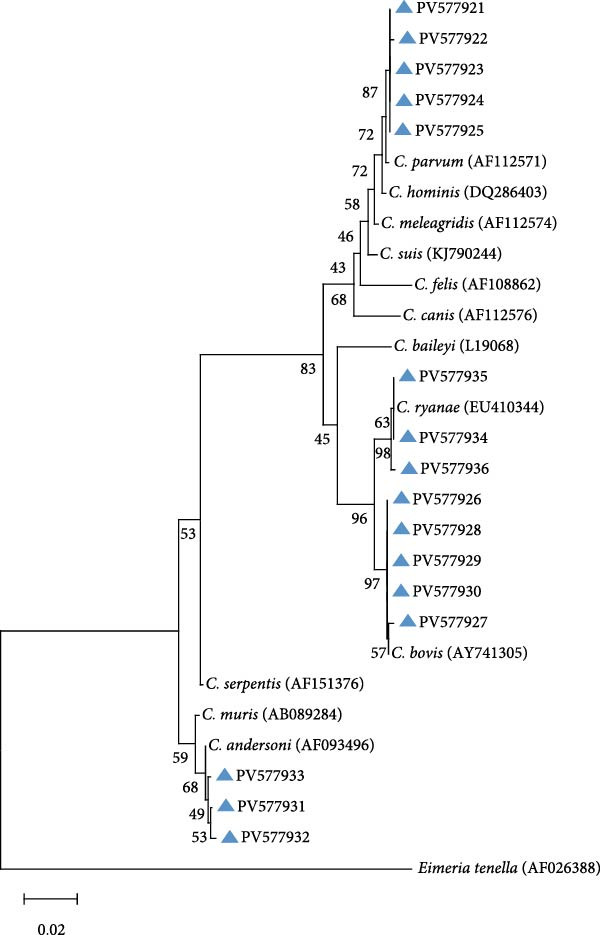
Phylogenetic relationships of *Cryptosporidium* spp. based on the maximum likelihood analyses of the *SSU* rRNA gene. Bootstrap values >50% from 1000 replicates are displayed. The blue triangles indicate sequences identified in the present study. The scale bar indicates 0.20 nucleotide substitutions per site.

Integrated RFLP profiling and *SSU* rRNA gene sequencing of PCR amplicons revealed four distinct *Cryptosporidium* species, including cases of interspecific coinfections (Table 1). Phylogenetic reconstruction further confirmed the taxonomic delineation of these species and uncovered substantial intragenotypic diversity within the *Cryptosporidium* population (Figure 2).

On the farms (Farm A, B, and C) of Jilin Province, *C. parvum* emerged as the predominant species, constituting 68.79% (97/141) of positive samples. Sequence analysis delineated four distinct genotypes within *C. parvum*: 40 isolates (PV577922) showed 100% identity to reference sequence MN557143, 24 isolates (PV577924) exhibited 100% identity to OR994136, 22 isolates (PV577923) matched PP838625 with complete identity, and six isolates (PV577921) shared 99% identity with OR363651, while five samples failed sequencing due to low DNA integrity. Among other species, *C. bovis* was detected in 23 isolates, with 10 isolates (PV577926) showing 100% identity to reference sequence OR364460, six isolates (PV577928) aligning perfectly with PP023939, five isolates (PV577930) demonstrating 100% identity to OP861724, and JX416365 and KC618608 each matching 100% with one sample (PV577927 and PV577929). *C. andersoni* was identified in eight isolates, of which six (PV577931) shared 99% identity with OP861848 and two (PV577932) matched KX926456 at 99% identity. All eight *C. ryanae* isolates (PV577935) displayed 100% identity to JN400880. Notably, five mixed infections were found, comprising three coinfections of *C. bovis* and *C. ryanae*, one dual infection involving *C. parvum* and *C. ryanae*, and one case of *C. bovis* and *C. parvum* coinfection.

On the Farm D of Heilongjiang Province, *C. parvum* was identified as the predominant species, including 61.54% (16/26) of positive samples, with all 16 isolates (PV577922) demonstrating 100% sequence identity to the reference sequence MN557143. One isolate (PV577935) was identified as *C. ryanae*, exhibiting 100% identity to reference sequence JN400880. Meanwhile, eight mixed infections were detected, including one case of *C. parvum* and *C. ryanae* coinfection, three instances of *C. bovis* and *C. ryanae* coinfection, and five coinfections involving *C. parvum*, *C. bovis*, and *C. ryanae*.

On the Farm E of Liaoning Province, *C. ryanae* predominated among positive samples, accounting for 58.06% (18/31), with sequence analysis revealing three distinct genetic profiles: 10 isolates (PV577935) exhibited 100% identity to reference sequence JN400880, four isolates (PV577934) matched KP793010 with complete identity, and four isolates (PV577936) aligned perfectly with reference sequence OQ456125. *C. bovis* ranked as the second most prevalent species (32.26%, 10/31), comprising seven isolates (PV577928) showing 100% identity to PP023939 and three isolates (PV577926) sharing 100% identity with OR364460. One *C. parvum* isolate (PV577921) was detected, demonstrating 99% identity to reference sequence OR363651. Additionally, two cases of *C. bovis–C. ryanae* coinfection were identified.

On the Farm F of Inner Mongolia, *C. parvum* was identified in 10 isolates, with sequence analysis revealing three distinct genetic profiles: eight isolates (PV577922) exhibited 100% identity to reference sequence MN557143, one isolate (PV577925) matched MK014775 with complete identity, and one isolate (PV577921) showed 99% identity to OR363651. *C. bovis* was detected in 10 isolates, demonstrating two genetic variants: six isolates (PV577926) shared 100% identity with OR364460, and four isolates (PV577928) aligned with PP023939 at 100% identity. Also, one *C. andersoni* isolate (PV577933) was confirmed, showing 100% sequence identity to reference strain KX710085.

Different geographical variation exists in *Cryptosporidium* species distribution (Figure 3A), with spatial factors being a key determinant of *Cryptosporidium* genotype prevalence patterns. Moreover, age‐stratified analysis revealed significant disparities in *Cryptosporidium* species distribution (Figure 3B). Preweaned calves exhibited the highest *C. parvum* prevalence (63.29%, 131/207), contrasting sharply with the 1.46% (3/205) detection rate in postweaned calves aged 3–11 months. *C. parvum* was absent in adult cattle (>1 years). *C. ryanae* and *C. bovis* infections predominated in calves aged 3–11 months, and *C. andersoni* was primarily detected in adult cattle (>1 years). Strikingly, coinfections involving multiple *Cryptosporidium* species were exclusively identified in cattle aged 3–11 months.

Figure 3The distribution of *Cryptosporidium* species across different regions (A) and age groups (B).

**Figure:**
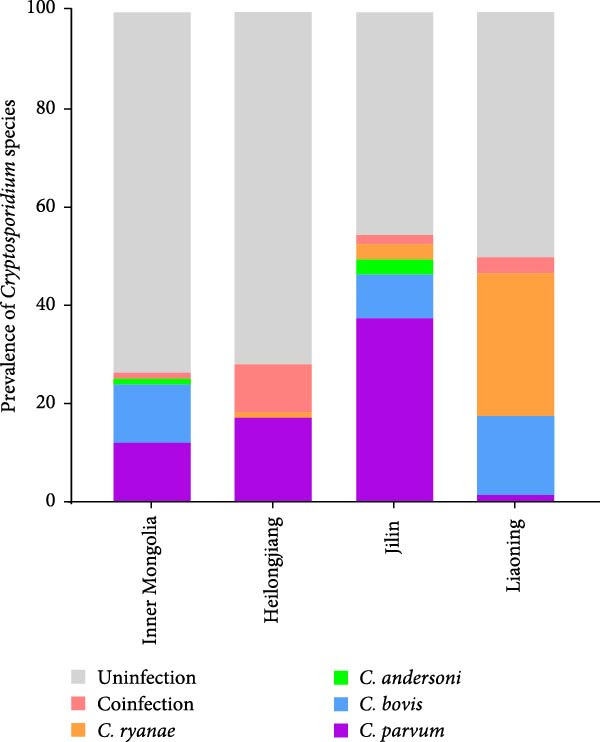
(A)

**Figure:**
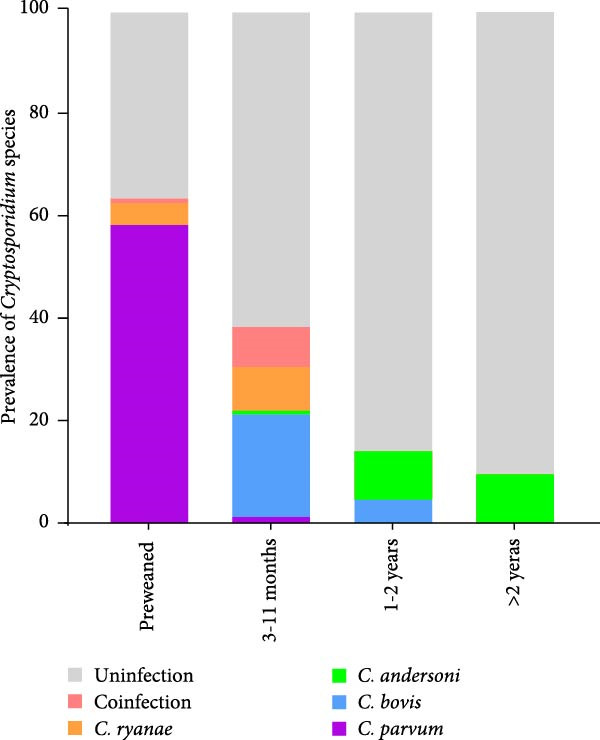
(B)

### 3.3. *C. parvum* Subtypes

All *C. parvum*‐positive samples were genotyped by sequencing the *gp60* locus via nested PCR, revealing a single genotype per farm (Table 3). Three distinct genotypes were identified: IIdA19G1 (detected in Farm A and B in Jilin, Farm E in Liaoning, and Farm F in Inner Mongolia), IIdA24G2 (exclusive to Farm C in Jilin), and IIdA21G1 (found solely in Farm D in Heilongjiang).

**Table 3:** Subtype of *C. parvum* in cattle in northeast China.

Subtype (No.)	Location (No.)	Farm ID	Source (this study)	Source (previous study)	Refs.
IIdA19G1	Jilin	Farm A (20) Farm B (37) Farm E (1) Farm F (10)	Cattle	Deer/sheep/horse/cattle/human	[48–52]
	Liaoning				
	Inner Mongolia				

IIdA24G2	Jilin	Farm C (40)	Cattle	Cattle/soil/water	[29, 53]

IIdA21G1	Heilongjiang	Farm D (16)	Cattle	Horse/sheep/human	[54–56]

### 3.4. *G. duodenalis* Assemblage A and E Subtypes

Amplification of the *bg*, *gdh*, and *tpi* gene loci confirmed the presence of *G. duodenalis* in all four regions (Table 4). Both PCR product sequencing and phylogenetic analysis were used for assemblage identification (Figure 4). Assemblages A and E were detected across all farms, with assemblage E representing the predominant assemblage. A total of 136 sequences were obtained from the *bg* locus, 122 from the *gdh* locus, and 84 from the *tpi* locus.

Figure 4Phylogenetic relationships of *G. duodenalis* based on the maximum likelihood analyses of the *bg* gene (A), *gdh* gene (B) and *tpi* gene (C). Bootstrap values >50% from 1000 replicates are displayed. The blue triangles indicate sequences identified in the present study. The scale bar indicates 0.20 or 0.02 nucleotide substitutions per site.

**Figure:**
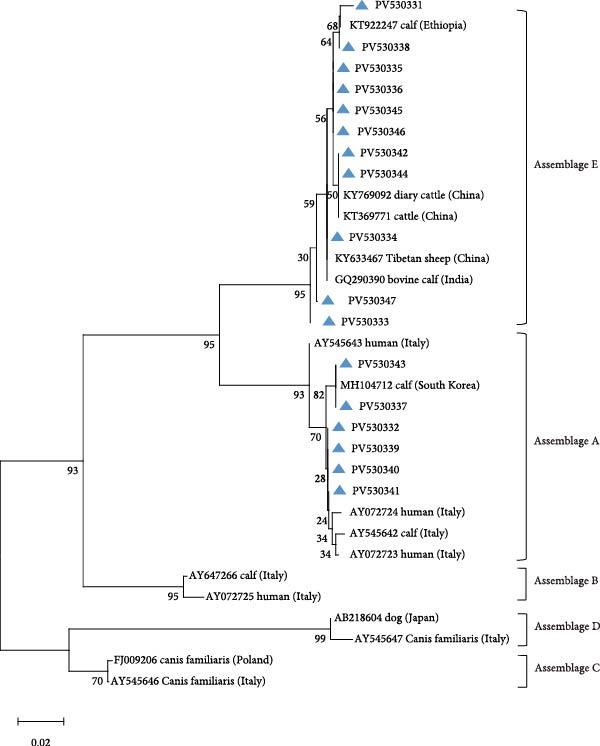
(A)

**Figure:**
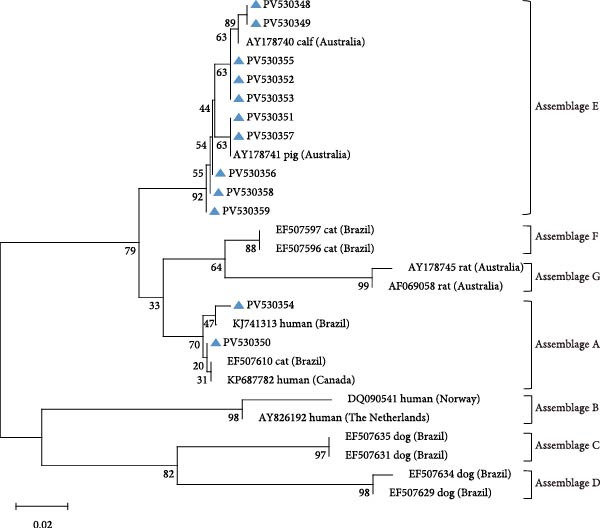
(B)

**Figure:**
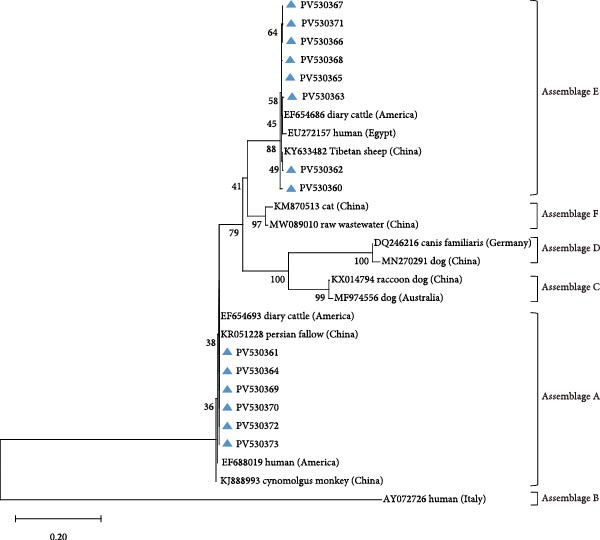
(C)

**Table 4:** Sequence variations at three genetic loci of *G. duodenalis* assemblage E in cattle in northeast China.

(a) Inter‐subtypic divergence in bg gene nucleotide composition within Assemblage E isolates											
Subtype (no.)	Nucleotide at position						GenBank accession number	No. positive			
	72	117	222	324	450	465		Jilin	Heilongjiang	Liaoning	Inner Mongolia
Ref. sequence	C	T	A	C	G	T	DQ116624				
E3 (31)						C	KP635114	17	0	2	12
E2 (30)							KP635113	17	0	5	8
E1 (30)				T			KT922247	6	1	19	4
E11 (9)			G			C	DQ116622	6	1	2	0
E5 (7)		C					KP334150	2	0	2	3
E8 (2)		C				C	KR075939	1	0	0	1
E6 (1)		C	G			C	EU726982	1	0	0	0

(b) Inter‐subtypic divergence in gdh gene nucleotide composition within Assemblage E isolates																	
Subtype (no.)	Nucleotide at position												GenBank accession number	No. positive			

	95	116	131	182	200	254	269	272	445	463	488	491		Jilin	Heilongjiang	Liaoning	Inner Mongolia
Ref. sequence	T	C	A	C	G	C	C	T	G	G	G	C	KF843923				
E3 (51)	C												KF843925	24	1	5	21
E1 (37)													KF843923	7	1	24	5
E21 (5)	C			T									KP334145	5	0	0	0
E11 (3)	C		G		A			G					KY710744	3	0	0	0
E14 (2)	C								A				KC960648	1	0	1	0
E29 (2)	C									A			KT369779	2	0	0	0
E2 (1)	C		G										KF843924	1	0	0	0
E40 (1)	C		G					G	A				MK645792	0	0	0	1

(c) Inter‐subtypic divergence in tpi gene nucleotide composition within Assemblage E isolates									
Subtype (no.)	Nucleotide at position				GenBank accession number	No. positive			
	98	114	335	501		Jilin	Heilongjiang	Liaoning	Inner Mongolia
Ref. sequence	C	G	T	G	DQ157270				
E11 (36)	T				KY769101	15	9	9	3
E3 (20)					DQ157270	8	3	3	6
E1 (4)		A			KT369761	1	1	2	0

Using DQ116624 as the reference sequence for the *bg* locus, seven previously reported genetic subtypes were identified: E3 (*n* = 31), E2 (*n* = 30), E1 (*n* = 30), E11 (*n* = 9), E5 (*n* = 7), E8 (*n* = 2), and E6 (*n* = 1).

The *gdh* locus was analyzed using KF843923 as the reference sequence, which led to the identification of six previously reported genetic subtypes: E3 (*n* = 51), E1 (*n* = 37), E21 (*n* = 5), E14 (*n* = 2), E29 (*n* = 2), and E2 (*n* = 1).

Reference sequence DQ157270 was employed for *tpi* locus analysis, resolving three documented subtypes: E11 (*n* = 36), E3 (*n* = 20), and E1 (*n* = 4).

For assemblage A, analysis of the *bg* locus identified 26 samples, comprising 14 A1 and 12 A2 subtypes. A1 exhibited 99% homology with reference sequences LC437420 (*n* = 10), KM926506 (*n* = 2), EU726988 (*n* = 1), and OM115992 (*n* = 1), while A2 showed 99% homology with EU642897 (*n* = 9), EU014386 (*n* = 2), and PP786683 (*n* = 1). At the *gdh* locus, only assemblage A1 was detected, demonstrating 99% homology with AY178735 (*n* = 16) and KF843930 (*n* = 4). Further analysis of the *tpi* locus resolved 22 A1 and 2 A2 subtypes: A1 aligned with EF654693 (*n* = 10), KR051228 (*n* = 6), JX845434 (*n* = 5), and KM926546 (*n* = 1) (99% homology), whereas A2 matched OM273020 (*n* = 2) (99% homology).

### 3.5. Multilocus Genotypes

A total of 41 samples were genotyped across the *bg*, *gdh*, and *tpi* loci, comprising 13 assemblage E MLGs, one assemblage A MLG, and eight mixed A + E infections (Table 5). Particularly, most MLGs exhibited region‐specific distribution, with only a few shared across regions, such as MLG‐E3, MLG‐E6, MLG‐E7, and Mixed 3 (A + E). MLG‐A1 (the sole assemblage A MLG) was exclusively detected in Inner Mongolia. Conversely, Inner Mongolia was the only region where no mixed infections were identified. Phylogenetic analysis showed all assemblage E MLGs clustered broadly with previously reported Tibetan sheep and yak isolates from Qinghai Province, northwest China (Figure 5A). The assemblage A MLG from one cattle isolate was identified as a solitary branch (Figure 5B).

Figure 5(A, B) Phylogenetic relationships of *G. duodenalis* MLGs. The neighbor‐joining tree was constructed using concatenated sequences of the *bg*, *gdh*, and *tpi* genes, based on genetic distances calculated with the Kimura‐2 parameter model. Bootstrap values >50% from 1000 replicates are displayed. The blue triangles indicate MLGs identified in this study.

**Figure:**
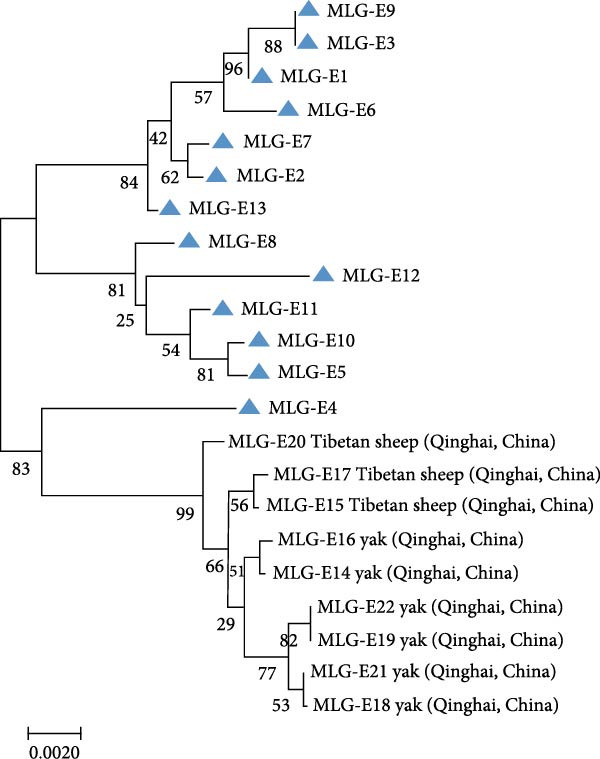
(A)

**Figure:**
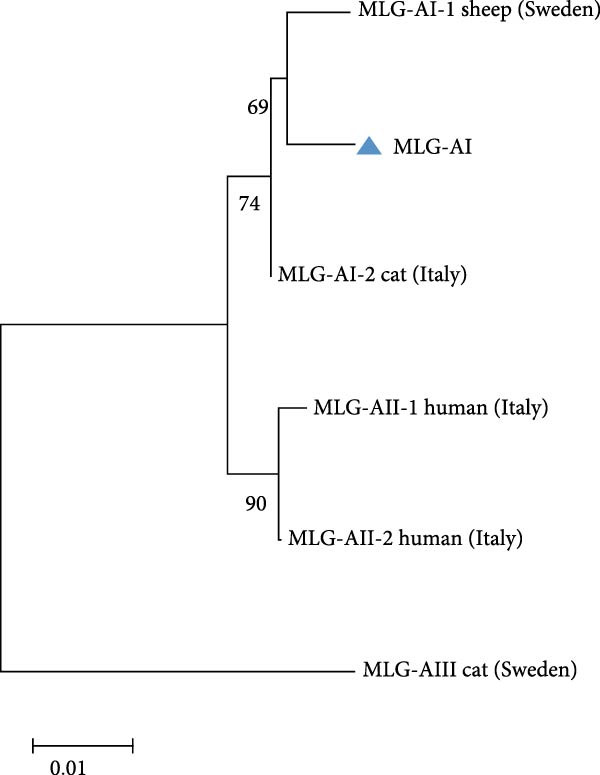
(B)

**Table 5:** Multilocus sequence genotyping of *G. duodenalis* in cattle in northeast China.

MLGs^a^	Genotype			Location (no.)
	*bg*	*gdh*	*tpi*	
MLG‐E1	E3	E3	E11	Jilin (1)
MLG‐E2	E1	E3	E3	Jilin (1)
MLG‐E3	E11	E3	E11	Jilin (1), Heilongjiang (1)
MLG‐E4	E3	E11	E11	Jilin (1)
MLG‐E5	E2	E1	E11	Jilin (1)
MLG‐E6	E2	E3	E11	Jilin (1), Liaoning (1)
MLG‐E7	E2	E3	E3	Jilin (3), Inner Mongolia (2)
MLG‐E8	E1	E1	E3	Heilongjiang (1)
MLG‐E9	E11	E3	E11	Heilongjiang (1)
MLG‐E10	E1	E1	E11	Liaoning (4)
MLG‐E11	E3	E1	E11	Liaoning (1)
MLG‐E12	E3	E1	E1	Liaoning (1)
MLG‐E13	E3	E3	E3	Inner Mongolia (2)
MLG‐A1	A1	A1	A1	Inner Mongolia (1)
Mixed 1 (A + E)	E8	E3	A1	Jilin (1)
Mixed 2 (A + E)	A2	A1	E11	Jilin (2)
Mixed 3 (A + E)	E2	E3	A1	Jilin (2), Liaoning (3)
Mixed 4 (A + E)	A1	A1	E1	Heilongjiang (1)
Mixed 5 (A + E)	A1	A1	E11	Heilongjiang (1)
Mixed 6 (A + E)	A1	A1	E3	Heilongjiang (2)
Mixed 7 (A + E)	E1	E1	A1	Liaoning (4)
Mixed 8 (A + E)	E2	E1	A1	Liaoning (1)

^a^The MLGs were named based on genotypes at *bg*, *gdh* and *tpi* loci.

**Figure 6:**
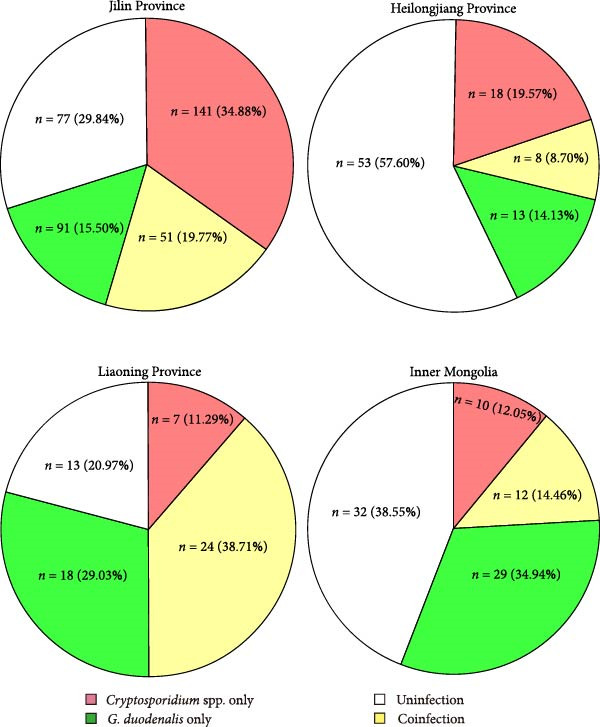
Proportions of single and coinfections of *Cryptosporidium* and *G. duodenalis* in four regions.

*3.6. Coinfection of C. parvum and G. duodenalis*. Among 495 samples of the study, 95 samples had coinfections of *C. parvum* and *G. duodenalis*. The rates of coinfection varied across regions: 19.77% in Jilin (*n* = 51), 8.70% in Heilongjiang (*n* = 8), 38.71% in Liaoning (*n* = 24), and 14.46% in Inner Mongolia (*n* = 12) (Figure 6).

## 4. Discussion

This study revealed a notably high prevalence of *Cryptosporidium* spp. and *G. duodenalis* infections on cattle farms in northeast China. The overall prevalence of *Cryptosporidium* was 44.44% (220/495; 95% CI 40.1–48.8), exceeding rates reported in Canada (27.30%, 39/143) [57], Italy (38.8%, 57/147) [58], and Estonia (23.0%, 112/486) [59]. Domestically, the prevalence was also higher than findings from Heilongjiang (4.4%, 40/909) [53], Shanxi (11.19%, 96/858) [60], Shandong (32.3%, 144/446) [61], and Henan (21.5%, 172/801) [62], though slightly lower than that observed in Xinjiang (48.7%, 185/380) [63]. Collectively, the *Cryptosporidium* infection rate identified in this study surpasses the average prevalence levels reported in regions across China [64]. This study identified a *G. duodenalis* infection prevalence of 39.39% (195/495), higher than rates reported in Turkey (30.2%) and Zambia (34.5%) [65, 66], yet lower than that of New Zealand (40.6%) [67]. Compared to previous domestic studies: Yunnan (10.49%) [68], Xinjiang (13.4%) [69], Hubei (22.6%) [70], Sichuan (41.2%) [71], Shanghai (60.1%) [72], and Guangdong (74.2%) [73], the *Giardia* infection rate identified here remains notably elevated within China.

Despite being located within the same province of Jilin, Farms A, B, and C exhibited marked variations in *Cryptosporidium* and *Giardia* infection rates, highlighting potential epidemiological heterogeneity even among geographically proximate cattle operations. Discrepancies in *Cryptosporidium* and *Giardia* infection rates across studies may stem from multifactorial causes, including methodological variations in detection techniques, age demographics of animal cohorts, heterogeneity in sample sizes, physiological status of hosts during sampling, timing of specimen collection, geographical and environmental factors, and farm management practices. Furthermore, studies indicate that *Cryptosporidium* oocysts and *Giardia* cysts exhibit environmental resilience, maintaining viability for extended periods under optimal temperature and humidity conditions [74, 75]. The comparatively lower temperatures in northern China may provide favorable conditions for their persistence, potentially contributing to the elevated infection rates observed in this study.

The age‐stratified distribution of *Cryptosporidium* species observed in this study is consistent with previous epidemiological reports [21, 23, 75, 76], demonstrating consistency in host–parasite dynamics across developmental stages of cattle. *C. parvum* predominates in preweaned calves; *C. bovis* and *C. ryanae* in weaned calves; while *C. andersoni* is age‐restricted to yearlings and adults. Interestingly, coinfections involving 2–3 *Cryptosporidium* species occurred predominantly in calves aged 3–11 months. This age‐specific pattern raises a critical mechanistic question: Is this phenomenon driven by the cessation of maternal antibody protection postweaning, or does it reflect a transitional phase in host susceptibility—shifting from *C. parvum* dominance to colonization by other *Cryptosporidium* species? This presents an intriguing question with epidemiological implications.

The previous study showed that *C. parvum* prevalence on surveyed farms increased from 26.0% (2011–2016) to 46.8% (2017–2021) [44]. *C. parvum* emerged as the predominant species in this study, particularly among preweaned calves, where its high prevalence correlates with impaired growth performance. Notably, *C. parvum* is a confirmed etiological agent of human cryptosporidiosis and demonstrated broad zoonotic potential across taxonomically diverse hosts, including rodents, canids, and ruminants [77]. Subtyping *C. parvum* isolates provides critical insights into host tropism and pathogen source tracking. Based on *gp60* locus analysis, three subtype families (IIa, IIc, and IId) are most prevalent among the ~20 identified *C. parvum* subtypes globally [78]. In Chinese dairy cattle, subtypes IIdA14G1, IIdA15G1, IIdA17G1, IIdA19G1, IIdA20G1, and IIdA24G2 have been previously documented [53, 78]. The present study identified three *gp60* subtypes: IIdA19G1, IIdA24G2, and IIdA21G1. The *C. parvum* subtype IIdA19G1 is the most epidemiologically widespread variant in Chinese dairy cattle and has also been detected in other livestock species, including deer, sheep, and horses [48–51]. Moreover, its recent identification in children and HIV‐positive patients in China underscores its zoonotic transmission potential [52]. The *C. parvum* subtype IIdA24G2 represents its third reported occurrence in China. It was first detected in dairy cattle herds in Heilongjiang [53], secondly identified in soil and pond water near cattle farms in Jilin which is different from the survey area of this study in Jilin [29]. The novel genotype IIdA24G2 has recently emerged as a prevalent strain in northeastern China. Subtype IIdA24G2 demonstrates high homology with IIdA24G1, differing by only a single TCG trinucleotide in their *gp60* sequences. Because IIdA24G1 has been previously reported in cattle from Sweden and Poland, as well as in diarrheic patients in the United Kingdom, IIdA24G2 is likely a potential zoonotic subtype capable of infecting both humans and multiple animal species. On the other hand, subtype IIdA21G1 has been previously identified in humans, sheep and horses [54–56]. It was noteworthy that subtype IIdA21G1a and IIdA21G1b were described in the previous studies. According to the *gp60* gene‐based nomenclature [21], GenBank Accession No. DQ280497 was designated as the reference sequence for IIdA21G1, and subtypes differing from this sequence are assigned subsequent alphabetical extensions. IIdA21G1a was found in human in Iran (AB560746) [79], and IIdA21G1b was described in cattle in Romania (KC469692) [80]. The sequences of IIdA21G1a and IIdA21G1b were found to contain trinucleotide repeats, yet their lengths were only half that of the DQ280497 sequence. Meanwhile, IIdA21G1b was observed to have two nucleotide differences compared to DQ280497. In this study, the sequences identified as IIdA21G1 were identical to that of DQ280497. To our knowledge, this represented the first confirmed detection of the IIdA21G1 subtype in cattle. The introduction of the novel genetic subtype has significantly expanded the diversity of circulating *C. parvum* strains in China. This genetic expansion raised concerns about potential cryptosporidiosis outbreaks in bovine populations and possible dissemination to other regions of the country. Moreover, the IIdA21G1 subtype could represent a threat to human health in China, given its prior detection in HIV‐infected patients in Portugal [54].

Consistent with previous findings, *G. duodenalis* infection prevalence exhibits a strong age‐dependent association in cattle [23, 70, 81]. The data showed that calves under 1 year of age were predominantly affected, with significantly higher susceptibility observed compared to older cattle. Specifically, the infection rate in preweaning calves (0–3 months) exceeded that of calves aged 3–11 months, a pattern corroborated by prior reports [23, 70]. However, the opposed result exists, as some studies have documented lower infection rates in this 3–11 month cohort relative to preweaning individuals [69, 81], which was consistent with the present study. Collectively, the age of 1 year appeared to serve as a critical threshold for susceptibility modulation.

Multilocus sequence analysis employing the MLG model was implemented to elucidate the epidemiological features of *G. duodenalis* across human and animal populations spanning multiple geographical regions, providing critical insights into its zoonotic transmission dynamics. Genetic characterization across three polymorphic loci revealed distinct MLG distributions: 13 unique MLGs were identified within assemblage E, while assemblage A exhibited a single MLG. Only four MLGs were detected in two regions, indicating a regional distribution of MLGs. Notably, eight novel MLGs were detected in hybrid assemblage A + E strains, demonstrating complex recombination patterns and potential zoonotic risk.

The study demonstrated that preweaned and postweaned calves constituted the principal epidemiological reservoirs for zoonotic genotypes of *C. parvum* and *G. duodenalis*. Both parasites were transmitted through an interconnected animal‐environment‐human pathway: infective oo/cysts excreted by infected animals contaminate environmental matrices, with subsequent human exposure occurring via inadvertent ingestion of viable propagules or mechanical transfer by flies [82]. Current therapeutic interventions against cryptosporidiosis and giardiasis remained substantially limited. For livestock operations, stringent prevention of pathogen introduction is imperative, as eradication became epidemiologically unattainable once these parasites became established within farm ecosystems. Farm managers were advised to develop comprehensive biosecurity protocols and implement regular staff training programs focused on infection control principles. Animal handlers must rigorously adhere to personal protective measures during calf‐rearing activities, including the use of designated protective gear and maintenance of strict hygiene practices to minimize occupational exposure risks.

Ecosystem health served as the cornerstone for the prevention and control of *Cryptosporidium* and *G. duodenalis*, by effectively blocking the environmental transmission pathways of these pathogens at their source. Compared to traditional chemical disinfection or medical interventions, ecologically oriented prevention strategies offered greater sustainability and cost‐effectiveness, particularly in resource‐limited regions. Future efforts should prioritize research on the ecological health nexus and advance the application of the “ecological immunity” concept in public health practices.

## 5. Conclusion

This study reported the high prevalence of *Cryptosporidium* spp. and *G. duodenalis* in cattle from northeastern China, with the identification of genetic subtypes. The discovery of the novel *C. parvum* subtype and mixed assemblage A and E infections of *G. duodenalis* suggested potential genetic recombination among divergent parasite strains, highlighting an increasing risk of public health. Further studies are required to monitor the epidemiological patterns and genetic diversity of these parasites, enabling the implementation of targeted control measures to mitigate public health impacts.

## Ethics Statement

All protocols in this study were approved by the Research Ethics Committee of Jilin University (SY202202104). Fecal samples were collected via rectal retrieval with minimal handling duration. Prior authorization from cattle farm management was obtained before sample collection.

## Conflicts of Interest

The authors declare no conflicts of interest.

## Author Contributions

**Qile Yu:** investigation, writing – original draft, methodology, data curation. **Sining Chen:** investigation, writing – original draft, methodology. **Xichen Zhang and Xiaocen Wang:** writing – review and editing, methodology. **Qi Zhao:** data curation, methodology. **Mengfei Xu:** methodology. **Jianhua Li:** methodology. **Pengtao Gong:** methodology. **Xin Li and Xu Zhang:** writing – review and editing. **Nan Zhang:** conceptualization, funding acquisition, writing – original draft, supervision. Qile Yu and Sining Chen contributed equally to this work and are co‐first authors.

## Funding

This work was supported by the National Key Research and Development Program of China (Grant 2023YFD1801001 and 2021YFF0702900) and the National Natural Science Foundation of China (Grant 31972704).

## Supporting Information

Additional supporting information can be found online in the Supporting Information section.

## Supporting information


**Supporting Information** Table S1: Primers and reaction conditions in the characterization of the SSU rRNA gene of Cryptosporidium spp., the gp60 gene of C. parvum and the bg, gdh, and tpi genes of G. duodenalis.

## Data Availability

All the data generated or analyzed during this study are included in this published article and its supporting information files. Nucleotide sequences obtained in the study were submitted to the National Center for Biotechnology Information (NCBI) GenBank database under the following accession numbers: PV577921–PV577936 for *SSU* rRNA gene, PV530314–PV530330 for the *gp60* gene, PV530331–PV530347 for the *bg* gene, PV530348‐PV530359 for the *gdh* gene, and PV530360–PV530373 for the *tpi* gene.
