# Supplementary material for: Genetic Characterization and Zoonotic Potential of Cryptosporidium spp. and Giardia duodenalis in Cattle From Northeast China
Source: Transbound Emerg Dis. 2025 Jul 24;2025:6148130. doi: 10.1155/tbed/6148130 (PMC13189501; doi:10.1155/tbed/6148130)
Supplement: Supplementary file 1 — Supporting Information Table S1: Primers and reaction conditions in the characterization of the SSU rRNA gene of Cryptosporidium spp., the gp60 gene of C. parvum and the bg, gdh, and tpi genes of G. duodenalis. [file TBED-2025-6148130-s001.docx]

**Table S1** Primers and reaction conditions in the characterization of the *SSU* rRNA gene of *Cryptosporidium* spp.，the *gp60* gene of *C. parvum* and the *bg*, *gdh*, and *tpi* genes of *G. duodenalis*.

| **Species** | **Gene** | **Primer Sequences (5'-3')** | **Amplicon size (bp)** | **Cycling conditions** | **Refs.** |
| --- | --- | --- | --- | --- | --- |
| *Cryptosporidium* spp. | *SSU* rRNA | F1: TTCTAGAGCTAATACATGCG  R1: CCCATTTCCTTCGAAACAGGA  F2: GGAAGGGTTGTATTTATTAGATAAAG  R2: AAGGAGTAAGGAACAACCTCCA | 830 | PCR1:  94℃/30s,55℃/30s,72℃/2min,30 cycles  PCR2:  94℃/30s,58℃/30s,72℃/1min,30 cycles | [1] |
| *C. parvum* | *gp60* | F1: ATAGTCTCCGCTGTATTC  R1: GGAAGGAACGATGTATCT  F2: TCCGCTGTATTCTCAGCC  R2: GCAGAGGAACCAGCATC | 860 | PCR1:  94℃/30s,52℃/30s,72℃/2min,30 cycles  PCR2:  94℃/30s,55℃/30s,72℃/1min,30 cycles | [1] |
| *G. duodenalis* | *bg* | F1: AAGCCCGACGACCTCACCCGCAGTGC  R1: GAGGCCGCCCTGGATCTTCGAGACGAC  F2: GAACGAACGAGATCGAGGTCCG  R2: CTCGACGAGCTTCGTGTT | 511 | PCR1:  94℃/30s,55℃/30s,72℃/1min,30 cycles  PCR2:  94℃/30s,55℃/30s,72℃/40s,30 cycles | [2] |
|  | *gdh* | F1: TTCCGTRTYCAGTACAACTC  R1: ACCTCGTTCTGRGTGGCGCA  F2: ATGACYGAGCTYCAGAGGCACGT  R2: GTGGCGCARGGCATGATGCA | 530 | PCR1:  94℃/30s,52℃/30s,72℃/1min,30 cycles  PCR2:  94℃/30s,60℃/30s,72℃/40s,30 cycles | [3] |
|  | *tpi* | F1: AAATIATGCCTGCTCGTCG  R1: CAAACCTTITCCGCAAACC  F2: CCCTTCATCGGIGGTAACTT  R2: GTGGCCACCACICCCGTGCC | 530 | PCR1:  94℃/30s,50℃/30s,72℃/1min,30 cycles  PCR2:  94℃/30s,50℃/30s,72℃/40s,30 cycles | [4] |

**Reference**

[1] Y. Y. Feng, Y. Ortega, G. S. He, P. Das, M. Q. Xu, X. C. Zhang, R. Fayer, W. Gatei, V. Cama, and L. H. Xiao, "Wide geographic distribution of *Cryptosporidium* *bovis* and the deer-like genotype in bovines," *Veterinary Parasitology* 144, no. 1-2 (2007): 1-9.

[2] M. Lalle, E. Pozio, G. Capelli, F. Bruschi, D. Crotti, and S. M. Cacciò, "Genetic heterogeneity at the beta-giardin locus among human and animal isolates of *Giardia duodenalis* and identification of potentially zoonotic subgenotypes," *International Journal for Parasitology* 35, no. 2 (2005): 207-213.

[3] S. M. Cacciò, R. Beck, M. Lalle, A. Marinculic, and E. Pozio, "Multilocus genotyping of *Giardia* *duodenalis* reveals striking differences between assemblages A and B," *International Journal for Parasitology* 38, no. 13 (2008): 1523-1531.

[4] I. M. Sulaiman, R. Fayer, C. Bern, R. H. Gilman, J. M. Trout, P. M. Schantz, P. Das, A. A. Lal, and L. H. Xiao, "Triosephosphate isomerase gene characterization and potential zoonotic transmission of *Giardia* *duodenalis*," *Emerging Infectious Diseases* 9, no. 11 (2003): 1444-1452.
